# Application of a Validated Innovative Smart Wearable for Performance Analysis by Experienced and Non-Experienced Athletes in Boxing

**DOI:** 10.3390/s21237882

**Published:** 2021-11-26

**Authors:** Tobias Menzel, Wolfgang Potthast

**Affiliations:** Institute of Biomechanics and Orthopaedics, German Sport University Cologne, 50933 Cologne, Germany; potthast@dshs-koeln.de

**Keywords:** instrumented sport equipment, boxing monitoring system, smart wearable, punch force, strike trajectory, biomechanical analysis

## Abstract

An athlete’s sporting performance depends to a large extent on the technical execution of the athletic motion in order to achieve maximum effectiveness in physical performance. Performance analysis provides an important means of classifying and quantifying athletic prowess in terms of the significant performance aspects of the sport to provide objective feedback. This study aimed to analyze technical execution in terms of punch trajectory, force, velocity and time, considering the expert-novice paradigm by investigating the technical execution of 31 experienced and non-experienced athletes for the four main punching techniques of the cross, jab, uppercut and hook strike. The kinetic and kinematic data were collected by means of a boxing monitoring system developed and validated for in-field use. The research revealed significant correlation for executed punching trajectory and punch force in intragroup comparison and significant differences in intergroup comparison. No significant differences were detected for punch velocity in either inter- or intra-group paradigms. This study, through use of the sensor system, aligns with the results of existing publications conducted in laboratory conditions, in the assessment of punch force, punch speed and punch time and thus extends the state of research by use of a smart wearable in field method.

## 1. Introduction

An athlete’s sporting performance depends to a large extent on the technical execution of the athletic motion in order to achieve maximum effectiveness in physical performance in attacking and defensive situations [1]. To this end, performance analysis provides a useful means of classifying and quantifying athletic prowess in terms of the significant performance aspects of a sport, to provide feedback to the athlete themself as well as their coaches. The gathered data can consequently be used to modify and optimize the athletes training and therefore their future performance [2,3,4,5]. Although professional performance analysis from a technical, biomechanical, physiological and psychological perspective is regularly applied in many sports, such as football, rugby, athletics or the rebound disciplines like tennis [1,2,3,4,6,7,8,9,10,11,12,13,14,15], there are few studies conducted in the sport of boxing that describe a comprehensive and sport-related performance analysis in this regard. In the past, various instruments have been developed and used in laboratory conditions to determine biomechanical impact parameters of different punching techniques. The instruments used range from water-filled punching bags to measure the change of fluid pressure [16], to ballistic pendulums [17,18], equipped punching bags or boxing dynamometers with acceleration sensors [19] or force transducers [20,21,22,23,24,25] to measure punch acceleration and force, without offering a comprehensive measuring tool.

The number of studies focusing on performance analysis becomes even more limited in the field of boxing and martial arts when considering the comparison between experienced and non-experienced boxing athletes.

The investigation of sport-relevant techniques and the comparison of performance characteristics between experienced and non-experienced athletes has far-reaching potential for understanding a sport and, in particular, to highlight competition-relevant performance characteristics which differentiate athletes of different sport-specific levels of experience [26,27]. These studies enable, among other factors, the investigation of technical execution of an athletic locomotion, imagery, anticipation and muscle activity patterns during the execution of a specific athletic movement [28,29,30].

Within the study object of the expert–novice paradigm, numerous investigations have attempted to identify the characteristics that define an expert compared to a novice and how the expert performs different technical characteristics in specific movements [16]. Reviewing existing literature on the comparison of experts and non-experts, it becomes apparent that in some disciplines, such as soccer, tennis or rugby, it highlights useful performance criteria for experienced and high-performance athletes [26,31,32,33,34,35,36,37,38].

However, this type of scientific research has so far found only limited application in the field of boxing and martial arts. Such studies focus primarily on the number of punches or the maximum force of punches thrown during a boxing match or sparring to conduct a comparison between the two groups of experienced and non-experienced athletes [29,39,40,41,42]. Analysis of the technical implementation and trajectory of the fist during a boxing punch in order to differentiate between the technical execution of experienced and non-experienced athletes is yet to be studied.

Predominantly in existing studies in the field of boxing sciences, the focus is on experienced athletes without discussion of athletes with less or no experience in the type of sport [14,43,44,45,46,47,48,49].

Based on this research gap, this study concentrates on the expert–novice paradigm with the goal of analyzing punching technique in experienced versus non-experienced boxers to identify characteristics of an expert athlete. Therefore, a specific focus is laid on the kinematic characteristics of the fist in three-dimensional space, starting from the defensive position, until the point of contact with the target and return, back to the defensive position. The objective of the study is to highlight the distinctive movement patterns performed by athletes with different levels of experience for the four main punching techniques: the straight cross, straight jab, the semi-circular uppercut and the hook punch [47]. The motion pattern is an important variable to analyze incorrect punch trajectories and the deviation from the ideal path of the fist for the individual punching techniques [50]. The experimental research investigates, in addition to the trajectory and orientation of the movement in three-dimensional space, the resulting punch force, punch speed and punch time separated into the three phases of the throw, contact and retraction period, between the two tested groups of experienced and non-experienced boxing participants.

Significant research has been undertaken in recent years on detecting human activity and the measurement of biomechanical performance parameters using portable sensor technologies, so-called wearable sensor technologies [51,52]. An innovative sensor system in the form of a smart wearable was developed to investigate the biomechanical punching characteristics presented in this paper [53]. The sensor system enables the instrumentation of a boxing glove for comprehensive data acquisition of kinetic as well as kinematic punch parameters such as punch force, punch time progression, punch velocity, acceleration, punch trajectory and much more.

Despite this, users remain largely unaware how reliable and accurate the data provided by wearable sensor technologies are [54]. This problem is due to the fact that from a scientific perspective, only a few wearable devices have been tested extensively to determine their accuracy, reliability and validity [55]. The lack of information about and need for the validity of developed wearable sensor technologies has been discussed by many authors [50,51,52,56,57,58,59].

In order to enable a scientifically correct application, the developed wearable system was extensively validated after its research and development phase with existing gold standard measurement systems, including a Kistler force plate and Vicon motion capture system [51,54,60,61]. The system was validated with accuracies of up to R^2^ = 0.99 [53].

The information obtained through this study offers further insights into the technical execution of experienced boxers and may provide specific technique training recommendations. As stated by McGarry et al. [1], technique effectiveness and efficiency are developed and established in comparison with the athlete’s performance by identifying an optimal technical model or reference criteria. Furthermore, this study illustrates the potential benefits of the use of advanced sport equipment to provide reliable augmented feedback necessary for athletes to improve [1,62] and overcome limitations on the accuracy with which coaches and trainers can retrieve and improve critical events within the scope of performance [1].

## 2. Materials and Methods

### 2.1. Participants

Thirty-one subjects in total participated in the present study. At the beginning of the experiment, the participants were divided into two groups according to their level of experience in boxing. This was followed by the division, based on their experience in boxing in years. As in the experiment by Lenetsky et al. [45], volunteers with at least three years of boxing experience were classified as experienced athletes and participants with less than three years of boxing experience were classified as non-experienced athletes to clearly distinguish between the two observation groups. The group of experienced athletes comprised 11 subjects (mean ± SD: age = 26.29 ± 4.54 years, height = 178.86 ± 6.57 cm, body mass 79.43 ± 9.31 kg and experience 7.43 ± 3.34 years), whereas the group of non-experienced athletes comprised 20 subjects (mean ± SD: age = 21.67 ± 2.46 years, height = 179.27 ± 9.76 cm, body mass 75.92 ± 8.15 kg and experience 0.36 ± 0.44 years) (Table 1). All participants were informed in advance of the data collection protocol as well as the risks and benefits of the experiment. Prior to the experimental testing, each participant was instructed with a boxing specific warm up for muscle activation as well as to become familiar with the setting and the equipment to be used for data acquisition.

### 2.2. Experimental Setup and Protocol

**Figure 1:**
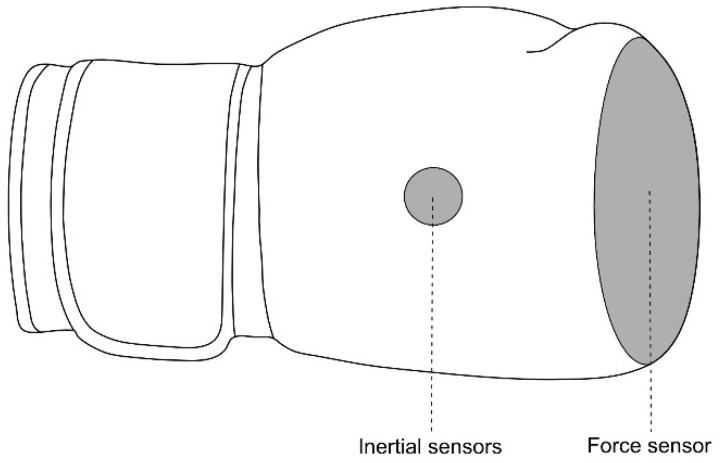
Schematic of the developed sensor system instrumented to the sport equipment.

This research used a developed and validated comprehensive punch performance sensor system on wearable sensor technology for the analysis of biomechanical parameters in the sport of boxing. The developed system consisted of force-sensing resistors, based on the piezoresistive principle, as well as a combination of acceleration, gyroscope and magnetic sensors for a comprehensive measurement of kinetic and kinematic boxing parameter. The sport equipment itself is defined by its size and weight as well as the materials used and is an integral part of the official competition regulations. In order to enable the instrumentation of the sport equipment without violating the official regulations, the instrumentation of the glove was made possible with the help of microtechnology and the development of customized flexible system components (Figure 1). This development allows the use of the latest sensor technologies without significantly changing the physical characteristics of the glove. Prior conducted validation experiments demonstrated the significant accuracies ranging from R^2^ = 0.97 to R^2^ = 0.99 of the sensor-derived measurements, in comparison to a force plate and Vicon motion capture system, for predicting boxing-specific biomechanical movement parameters while punching in field use [52].

To analyze punching technique in experienced versus non-experienced boxers, the subjects were instructed at the beginning of the study on the course of the experiment and the punching techniques to be thrown. This was to avoid misinterpretation of the punching techniques by the group of the inexperienced boxing participants.

The kinetic and kinematic data collection by means of the monitoring system included the measurement of punch force, punch acceleration, punch speed, fist trajectory and orientation in three-dimensional space as well as the punch time, separated into the throw, contact and retraction time.

The data acquisition was conducted using the aforementioned boxing glove monitoring system. The boxing monitoring system was instrumented into a 12 ounce (340.194 g) AIBA certified, 2017 model, boxing glove from Adidas (Adidas AG, Herzogenaurach, Germany) for each subject for data collection purposes. A 40 kg punching bag made out of leather from Paffen Sport (Paffen Sport GmbH & Co. KG, Cologne, Germany) was used on a wall-mounted suspension to perform the punches against a defined and stationary target.

The data acquisition of the boxing monitoring system was conducted with a data acquisition frequency of 1000 Hz and stored in a buffer to allow a comprehensive post processing and analysis. The high measuring frequency of 1000 Hz was selected to ensure that the entire punch course, including the throw, impact and retraction, is recorded for all kinetic and kinematic stroke parameters to be collected.

The experimental protocol consists of four punching techniques to be executed by all participants as the most used techniques in boxing [40]. To carry out the impact tests, the test subjects were instructed to perform the impacts with two different strike intensities with the help of a defined survey protocol. Each intensity was thrown five times. The study focused on the kinetics and kinematics of the punches thrown on the suspended boxing bag. The punches were accomplished by all participants starting in a static defense positioning facing the boxing bag as the target to be hit. At the beginning of each punching technique, the test subjects were encouraged to determine and test their own punch distance. Initially, the first intensity of each type of stroke was performed slowly with a special focus on technique performance. Subsequently, the subjects were instructed to perform the test with full effort, i.e., a maximum of 100% punch intensity.

The punch still must be executed with a technique close to competition in respect to time, as a decisive criterion of a successful punch is the duration of the punching time. This criterion is especially important in sparring or real competition situations, as strokes that take a long time to execute allow the opponent more time to react to the attack. The opponent may have a reduced reaction time for a quickly executed punch and therefore a lower chance to block the punch or even to execute a counterattack. This was to avoid strokes executed beyond the realistic punching technique used in sparring or competition.

After performing the punch, the test participants were instructed to return immediately to the defensive position, as in a sparring or competition scenario, to protect themselves against counterpunches. The subjects were instructed to remain in their defensive position for at least two seconds before the consecutive punch had to be performed.

**Figure 2:**
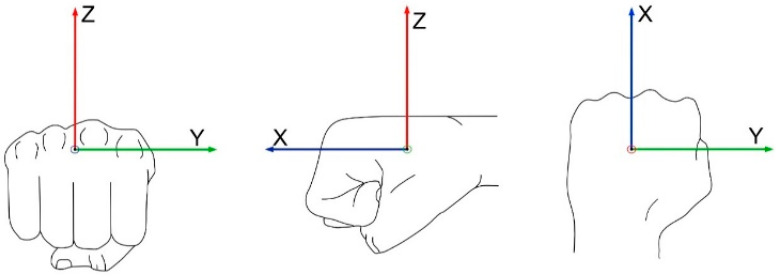
Body coordinate system of the boxer’s fist (X: anterior positive, posterior negative (rotation around the sagittal axis); Y: medial positive, lateral negative (rotation around the transverse axis); Z: dorsal positive, palmar negative (rotation around the longitudinal axis).

The coordinate system for the three-dimensional measurement in space was defined as illustrated in Figure 2. The acceleration in *x*-axis is pointing in punch direction (anterior positive, posterior negative), the *y*-axis to the medial and lateral side (medial positive, lateral negative) and the *z*-axis in the direction of the palm (dorsal positive, palmar, negative).

### 2.3. Data Analysis

The biomechanical performance data collected and buffered during the experimental execution of the punching tests were processed for further data handling and advanced data analysis using custom-built MATLAB (2018b) (The MathWorks, Natick, MA, USA) routines.

The data analysis of the defensive position was normalized for each subject individually. Therefore, the trajectory and orientation in three-dimensional space of the stroke was determined from the defensive position taken at the start of the first punch thrown. On this basis, the deviations of the defensive position for the following performed strikes were analyzed. This procedure was executed for all of the tested punching techniques. Rotations and movements in three-dimensional space were analyzed in terms of absolute angular rotations in degrees and motion trajectories in centimeters, starting from the subject’s prior determined defensive position.

The punch time was normalized in order to analyze the strike pattern of the thrown punching techniques to each other as well as among all participated subjects, based on the standardized sampling frequency of 1000 Hz. The absolute punch time was divided into the three phases of ‘attack’, ‘contact’ and ‘retraction’ back to the defensive position. The attacking time was determined from the initial movement of the fist in the direction of the striking object in the *x*-axis and finished by the first contact with the target to be hit. The contact phase was defined as the period in which the glove is in contact with the target to be hit. This phase was further divided into the exposure time until maximum compression at the targeting object, up to the maximum achieved impact force was achieved and the pre-release phase until the hand is released from the target. The retraction time was measured starting with the release of the fist from the object to be hit until the return to the defensive position and a reduced acceleration of the fist was finalized. Furthermore, the fist velocity, peak force, punch impulse and punch trajectory were measured and analyzed in three-dimensional space to compare the punching techniques of experienced and non-experienced athletes.

### 2.4. Statistical Analysis

The statistical analysis was conducted using the analysis software, IBM SPSS Statistics for Windows, Version 23.0 (IBM Corporation, New York, NY, USA).

The technical movement profiles between experienced and inexperienced boxers were calculated and compared as mean and standard deviation (SD) for each of the four punching techniques performed.

Due to the greater power of expression, the Shapiro–Wilk test was used in preference to the Kolmogorov–Smirnov test for the analysis of normal distribution. A three-way ANOVA was used to evaluate group differences. The individual differences between the two groups of participants as well as punching techniques were analyzed by means of a Tukey or Games–Howell post hoc test if the homogeneity of variances was not fulfilled. The check of homogeneity of the error variances was performed by the Levene Test (*p* > 0.05). The 95% confidence intervals were calculated with an alpha level set of *p* < 0.05 to verify statistical significance.

## 3. Results

**Figure 3:**
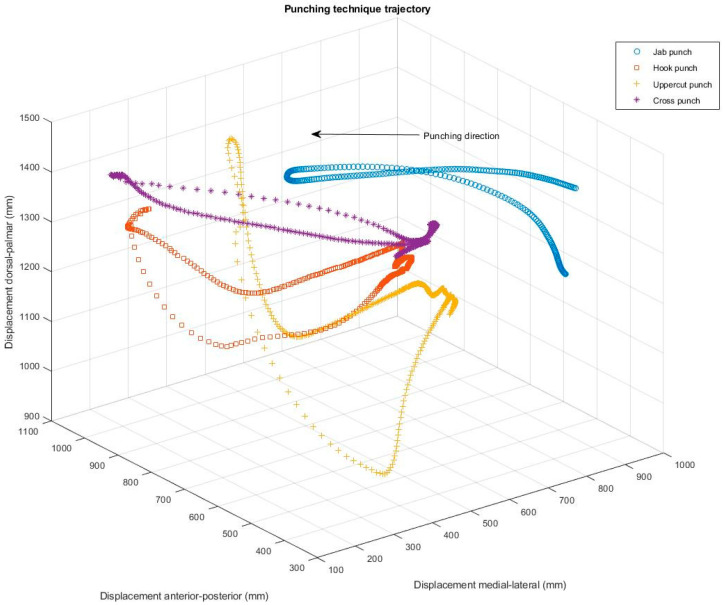
3D displacement graph of single punches performed against a boxing bag.

Figure 3 presents the trajectory of the fist from the defensive stance to the punching object of the punching bag and return to the defensive stance of the four punching techniques performed throughout the experimental study. The figure shows a clear distinction between the punching techniques tested with regard to displacement in three-dimensional space. The straight punching techniques of the jab and cross punch are executed in a straight line along the anterior–posterior sagittal plane (*x*-axis). The hook punching technique, on the other hand, shows a semicircular striking movement in a lateral direction on the transverse plane around the sagittal axes (*z*-axis). Whereas the second semicircular punching technique of the uppercut is performed in a semicircular movement around the horizontal axes (*y*-axis) from anterior to posterior (Figure 3).

The conducted three-way ANOVA showed a statistically significant difference for the overall analysis between the two groups of experience level F(21.00, 51.00) = 3.221, *p* < 0.001, partial η^2^ = 0.570, Wilk’s Λ = 0.430; the punching techniques performed F(63.00, 153.076) = 11.725, *p* < 0.001, partial η^2^ = 0.827, Wilk’s Λ = 0.005; and for the interaction between the expert level and punching techniques thrown F(63.00, 153.076) = 1.550, *p* = 0.016, partial η^2^ = 0.388, Wilk’s Λ = 0.229.

A detailed presentation of the results for the different stroke types of the two subject groups is presented in the subsequent sections.

### 3.1. Cross Punch Results

The first punching technique tested was the cross. Similar to the jab, the cross punch is a straight punch. In contrast to the jab (leading hand), the cross is performed by means of the strong striking hand.

The data sets of both groups of subjects showed a normal distribution of the data with *p* > 0.05.

The comparison of the initial fist position shows that the defensive position of the subjects of the experienced testing group take their fist in an average rotation of 62.68° (SD = 5.23°) around the transverse axis with a supination of 108.32° (SD = 16.57°) in the sagittal axis towards the target. The initial defensive position of the group of non-experienced subjects differed in comparison with a rotation of 5.81° in the transverse axis (95% CI [−3.02°, 14.64°]) and −4.88° in the sagittal rotation (95% CI [−25.54°, 15.77°]). This represents a mean defensive position of the inexperienced athletes with a rotation of 56.87° (SD = 16.11°) in the transverse axis, as well as a supination of 113.2° (SD = 22.46°) in the sagittal axis. No statistically significant difference in the defensive position between experienced and inexperienced subjects was detected in the rotation of the fist.

**Figure 4:**
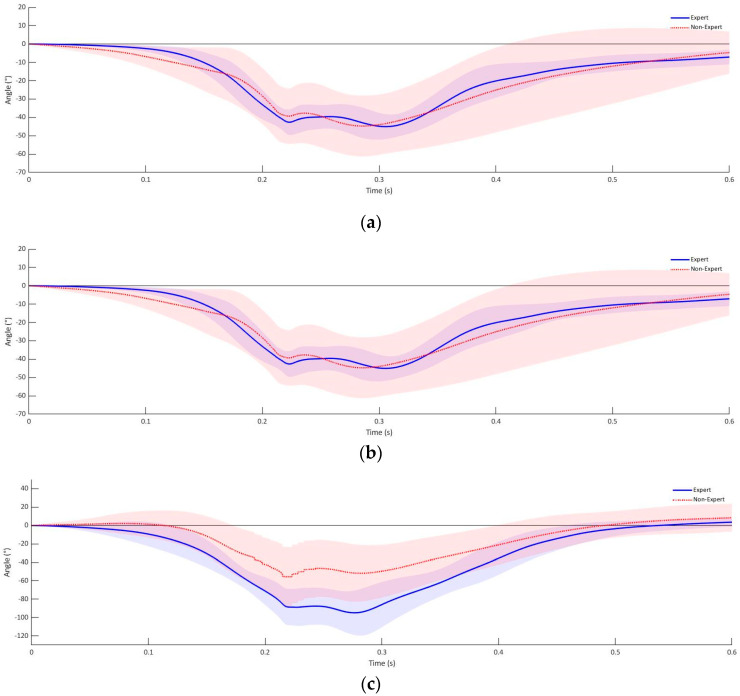
Fist rotation experienced athletes cross punch: (**a**) longitudinal, (**b**) transversal and (**c**) sagittal axis.

As demonstrated in Figure 4, it becomes apparent that the orientation of the fist to the object to be punched is initiated with a rotation around the longitudinal axis before the fist is orientated in the direction of the object to be hit in the transverse and sagittal axis. During the contact of the fist with the object to be struck, a mean rotation of 0.15° (SD = 13.27°) in the longitudinal axis is seen, compared to the initial defensive position of the experienced athletes. The rotation in the longitudinal axis at the time of the fist impact was higher for the group of inexperienced athletes with −7.81° than for the group of experienced athletes (95% CI [−13.61°, 29.23°]). Following the start of the rotation in the longitudinal axis, the fist of the experienced group of subjects is rotated by an average of −42.97° (SD = 3.1°) in the transversal axis and −86.21° (SD = 4.7°) in the sagittal axis up to the moment of contact with the object of impact (Figure 4). In comparison, the group of inexperienced athletes performed a rotation around the transverse axis of −39.75° (Figure 4) (SD = 10.41°) and a pronation of 59.41° (SD = 21.49°) in the sagittal plane until a first contact with the target (Figure 4). This corresponds to a mean difference of −3.22° (95% CI [−8.81°, 2.37°]) in the transversal axis and 26.81° (95% CI [15.84°, 37.78°]) in the sagittal axis between the inexperienced and experienced group of test subjects. The results of the rotations around the longitudinal and transverse axis from the initial defensive position to the impact of the fist on the striking object showed no statistically significant differences between the experienced and non-experienced group of test subjects, rotation around the longitudinal axis (*p* = 0.45) or rotation around the transverse axis (*p* = 0.24). A significant difference between experienced and non-experienced subjects was detected in the rotation around the sagittal axis from the defensive position to the initial contact (*p* < 0.001).

After impact, the fist is immediately returned to the defensive position. Table 2 shows a mean deviation of the orientation of the fist in three-dimensional space from the initial to the retracted position of −4.24° (SD = 3.85°) in the longitudinal axis, −1.92° (SD = 4.33°) in the transverse axis and −0.17° (SD = 6.42°) in the sagittal axis of the experienced group of participants. In the comparison of the experienced athletes, the group of non-experienced subjects presented a deviation of rotation in the longitudinal axis between the initial and retracted position of −4.95° (SD = 17.36°), a deviation of −2.51° (SD = 7.79°) in the transverse rotation and a deviation of 6.1° (SD = 14.93°) in the sagittal rotation. No statistically significant differences were tested between the initial and retracted positions of experienced and non-experienced athletes with respect to fist orientation in three-dimensional space.

The absolute punching time was defined as the time from the initial fist movement from the defensive position to the object to be punched and back to the defensive position. As forementioned, the entire punch was separated into the three phases of fist movement. The first phase was defined as the throwing phase. The throwing phase was defined as the time from the initial defensive position to the first contact with the target object. The second phase was defined as the contact phase. The contact phase is defined from the first contact of the fist with the punching object until the point of time, the glove is released from the punching bag. The third and, therefore, final phase started with the beginning of the release of the glove from the punching object back into the defensive position and was defined as the retraction phase.

The absolute punch time of the cross-punch technique was on average 402 milliseconds (SD = 65 ms) for the group of experienced athletes. With an average difference of −47 milliseconds (95% CI [−150.87, 55.55]), the total cross punch time for the inexperienced group was 450 milliseconds (SD = 104 ms). The first of the three defined movement phases of the fist, from the defensive position to the object to be punched, took 111 milliseconds (SD = 41 ms) in the experienced group of test persons, compared to 102 milliseconds (SD = 37 ms) in the inexperienced group of subjects. This resulted in a mean difference of 9 ms (95% CI [−31.04, 48.96]). From the first contact of the boxing glove with the object to be hit until the fist is released, the fist remains for 122 milliseconds (SD = 18 ms) in contact with the boxing bag for the expert group and 118 milliseconds (SD = 25 ms) in the group of non-experts. The punch is completed with the third phase of the fist movement back into the defensive position. This action phase averages 169 milliseconds (SD = 41 ms) in the expert group and 235 milliseconds (SD = 79 ms) in the non-experienced group of subjects. The statistical investigation revealed no statistically significant differences between the experienced and non-experienced group of subjects in the absolute impact time (*p* = 0.35) as well as the three temporal action phases of the throw (*p* = 0.65), contact (*p* = 0.72) and the retraction phase (*p* = 0.09) for the cross.

### 3.2. Hook Punch Results

After testing the cross, the hook technique was performed as the first semicircular punch. The detailed examination of the normal distribution using the Shapiro–Wilk test showed a normal distribution for the datasets of the experienced and inexperienced test groups with *p* > 0.05.

The defensive position of the experienced group of test persons measured at the beginning of each stroke showed an average rotation around the transversal axis of 58.03° (SD = 6.23°) and a pronation of 113.86° (SD = 30.47°) of the orientation of the fist in three-dimensional space. With an average difference of 15.83° in the transverse axis (95% CI [5.84°, 25.82°]) and 13.82° in the sagittal axis (95% CI [−23.35°, 51°]) the average defensive position of the inexperienced group of subjects was measured with a rotation of 42.2° (SD = 17.35°) in the transverse axis and 100.04° (SD = 17.8°) in the sagittal axis. This result showed a statistically significant difference in the defensive position of the transverse axis (*p* = 0.004), but no statistically significant difference in the orientation of the sagittal axis between experienced and inexperienced boxing subjects.

**Figure 5:**
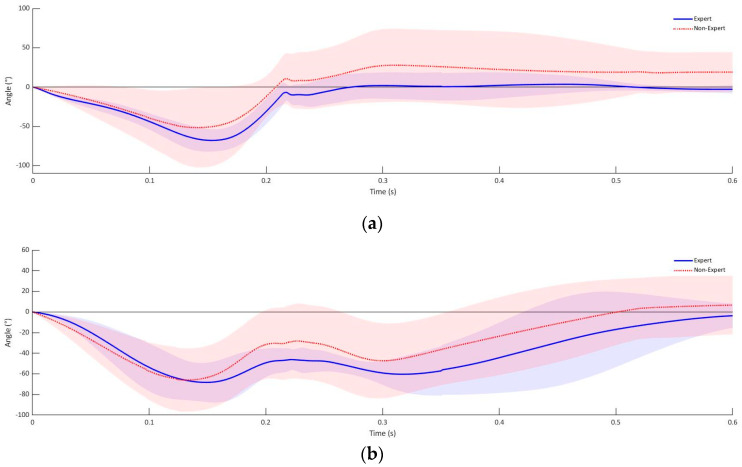

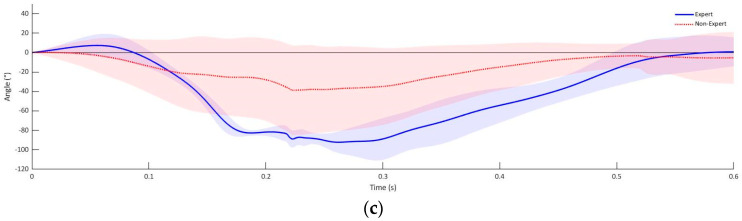
Fist rotation experienced athletes hook punch: (**a**) longitudinal, (**b**) transversal and (**c**) sagittal axis.

The rotation of the fist orientation in three-dimensional space shown in Figure 5 shows that the fist moves towards the target object with an average rotation of −70.94° (SD = 14.06°) around the longitudinal axis. At the time the fist reaches the target object, the longitudinal axis is rotated with an average of −11.54° (SD = 8.59) in the group of experienced subjects (Figure 5). A similar movement pattern is shown by the group of inexperienced participants in the rotation around the longitudinal axis from the defensive position to the point the fist makes contact to the target. The non-experienced group of participants performed the rotation in the longitudinal axis, with a laterally directed rotation of −51.58° to the target. This corresponds to a mean difference of 19.36° (95% CI [−10.95°, 23.65°]). At the target, the fist shows a −13.89° (SD = 17.98°) rotation compared to the defensive position in the longitudinal plane (Figure 5). In the transversal axis, the experienced group of test subjects tilted the fist by an average of −48.99° (SD = 8.21°), as well as a pronation in the sagittal axis of −79.38° (SD = 1.66°) at the point where the fist arrives at the target (Figure 5). In contrast, the group of inexperienced test subjects showed an inclination of the fist in the transverse axis of −35.88° (SD = 17.73°), as well as a rotation in the sagittal axis of −34.95° (SD = 22.14°) from the defensive position to the target (Figure 5). This corresponds to a mean difference of −13.11° in the transverse axis (95% CI [−24.66°, −1.57°]) and −44.29° in the sagittal axis (95% CI [−54.88°, −33.98°]). The rotation of the fist from the defensive position to the punching object around the longitudinal axis shows no statistically significant group difference between experienced and inexperienced boxers (*p* = 0.45). A statistically significant difference was analyzed between the experienced and non-experienced group of subjects in the rotation around the transverse axis (*p* = 0.02) as well as the sagittal axis (*p* < 0.001).

After the target has been hit, the fist is immediately returned to the defensive position for defensive purposes. The group of experienced boxing participants showed a mean deviation of the orientation of the fist in the three-dimensional space between the defense position before and after the impact of −9.78° (SD = 7.16°) in the longitudinal axis, −0.26° (SD = 2.45°) in the transverse axis and −4.56° (SD = 12.38°) in the sagittal axis (Table 3). The non-experienced group of subjects returned the fist to the defensive position following the executed punch with a mean deviation of −25.2° (SD = 30.94°) in the longitudinal axis, 7.10° (SD = 19.62°) in the transverse axis and −2.26° (SD = 23.1°) in the sagittal plane for the executed punches (Table 3). The deviation in the defensive position before and after the executed stroke showed no statistically significant differences in the defensive positions within a group of subjects, nor in the deviation between the experienced and inexperienced group of participants.

The analysis of the three defined impact phases for the hook punch shows that the absolute impact time was performed faster in the group of experienced subjects with an average duration of 441 milliseconds (SD = 104 ms) as compared with the group of non-experienced subjects whose average duration was 479 milliseconds (SD = 117 ms), with an average difference of 38 ms (95% CI [−169.07, 93.4]). The throw phase took an average of 91 ms (SD = 50 ms) in the experienced group of subjects and 72 ms (SD = 233 ms) in the inexperienced group. This corresponds to a mean difference of 18 ms (95% CI 119.01, −228.63]). In the second phase, the experienced group of test persons had 141 ms (SD = 29 ms) of contact with the object to be punched, from the first impact of the fist to the release of the punching bag. With an average difference of 71 ms (95% CI [−280.01, 138.07]), the fist of the inexperienced test persons was in contact with the object to be punched with a mean time of 212 ms (SD = 198 ms). The retraction phase of the fist from the target to the defensive position lasted on average 168 ms (SD = 97 ms) in the experienced group compared to the inexperienced group with 186 ms (SD = 83 ms). This corresponds to a mean group deviation of 18 ms. The investigation of group differences regarding the temporal movement phases of the fist shows no statistically significant differences in the absolute punch time (*p* = 0.55) nor in the three temporal action phases of the throw (*p* = 0.88), contact (*p* = 0.49) and the retraction phase (*p* = 0.68).

### 3.3. Jab Punch Results

As the third punch technique, the jab was performed. Similar to the cross, the jab is a straight punching technique. In contrast to the cross, the jab punch technique is performed with the leading hand and serves primarily as a punch to keep the opponent at a distance and prepare for a following effective punch.

The experienced group of participants showed a mean rotation of 58.25° (SD = 2.54°) in the transverse axis and a supination of 111.19° (SD = 27.84°) of the fist at the start of the test series as well as prior to each test cycle in the assumed defensive position. With an average difference of −4.55° in the transverse axis (95% CI [−10.39°, 1.29°]) and −8.9° in the sagittal axis (95% CI [−36.2°, 18.72°]) the inexperienced subjects took up the defensive position with a rotation of 62.81° (SD = 11.2°) in the transverse axis and a supination of 120.18° (SD = 30.12°). The orientation of the fist in three-dimensional space in the defensive position of experienced and non-experienced athletes showed no statistically significant differences.

**Figure 6:**
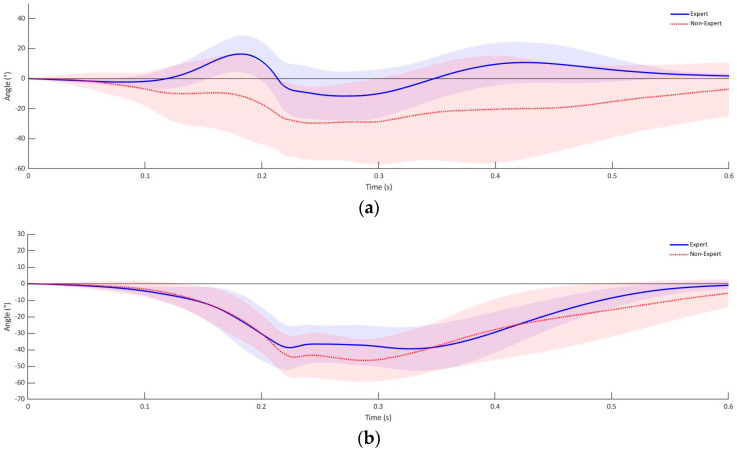

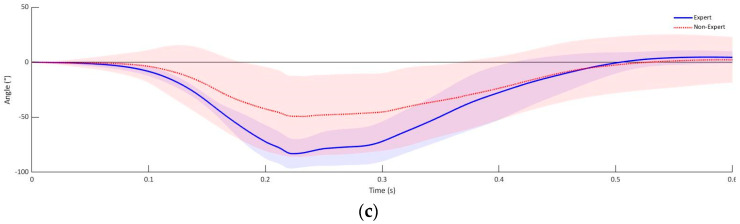
Fist rotation experienced athletes jab punch: (**a**) longitudinal, (**b**) transversal and (**c**) sagittal axis.

The movement of the fist towards the target object begins with a rotation in the longitudinal axis (Figure 6). This movement is followed by a temporally offset alignment of the fist around the transversal and sagittal axis (Figure 6). At the moment of the fist hitting the targeting object, the fist was rotated from the defensive position by an average of −11.75° (SD = 11.17°) in the longitudinal axis in the experienced group of test persons (Figure 6). With an average difference of 12.97° (95% CI [−10.31°, 28.26°]) the non-experienced group of subjects shows an average rotation of −24.72° (SD = 23.42°) in the longitudinal axis (Figure 6). The transversal rotation, which starts after the initial rotation in the longitudinal axis, had an average of −41.59° (SD = 3.12°) for the experienced group of test persons and −43.38° (SD = 13.92°) for the non-expert group until the fist hits the punching bag (Figure 6). This corresponds to a mean deviation of −2.21° (95% CI [−7.47°, 7.04°]) between the two tested groups. The third rotation in the sagittal axis shows a mean difference of the fist from the defensive position to the target of −36.16° (95% CI [−51.75°, −20.56°]) between the experienced test group (−82.2° (SD = 8.48°)) and the non-experienced test subjects (−46.04° (SD = 28.87)) (Figure 6). The rotation around the longitudinal and transverse axis from the defensive position to the object showed no statistically significant difference between the two tested groups’ rotation around the longitudinal axis (*p* = 0.35) and rotation around the transverse axis (*p* = 0.97). In contrast to the first two rotations, the rotation around the sagittal axis showed a statistically significant difference between the two groups in the rotation from the defensive position to the first contact with the target (*p* < 0.001) (Figure 6).

As shown in Table 4, a deviation of the fist orientation in three-dimensional space of 6.14° (SD = 8.47°) in the longitudinal axis, −6.16° (SD = 4.3°) in the transverse axis and 1.4° (SD = 2.8°) in the sagittal axis is shown between the first defensive position before the punch is executed to the defensive position after the impact was executed for the group of experienced participants. With an average difference of 8.34° to the experienced group, the retracted defensive position of the non-experienced group of subjects is set with a deviation of −2.2° (SD = 15.43°) from the initial defensive position (Table 4). In addition, the retracted defensive position deviates from the initial position by −9.63° (SD = 11.47°) in the transverse axis and −7.24° (SD = 18.34°) in the sagittal axis (Table 4). This corresponds to a mean difference from the experienced group by 3.47° in the transverse axis and 8.64° in the sagittal axis. The results presented do not show statistically significant differences between the two groups of subjects.

The investigation of the duration of the three defined impact phases for the jab punch technique shows a mean difference between the experienced and inexperienced group of test persons of 39 ms (95% CI [−44.71, 122.02]). The group of non-experienced subjects exhibits a shorter average duration of 485 ms (SD = 98 ms) than the experienced group, with an average duration of 524 ms (SD = 63 ms). In contrast to the total punch time, the phase of the throw was performed with a duration of 117 ms (SD = 25 ms). This shows a mean difference of −18ms (95% CI [−53.2, 17.67]) for the experienced group of test subjects compared to the inexperienced group with a duration of 135 ms for the throw. The fist of the inexperienced test persons exerts pressure on the punching bag with a mean contact time of 138 ms (SD = 28 ms). The retraction phase was measured with a duration of 212 ms (SD = 75 ms). With a difference of 6 ms (95% CI [−19.11, 31.8]), the third punching phase in the experienced group of test persons measured a duration of 144 ms (SD = 27 ms), while the retraction phase for the return to a defensive position took a mean 262 ms (SD = 42 ms). This corresponds to a mean difference of 50 ms in the third stroke phase between the two tested groups of participants. The investigation for significance shows that no statistically significant difference was measured for the total punch time (*p* = 0.35) as well as the first two defined movement phases of the throw (*p* = 0.31) and contact period (*p* = 0.61). In contrast, a statistically significant difference between the two tested groups was measured for the retraction phase with (*p* = 0.04).

### 3.4. Uppercut Punch Results

The fourth and last performed punching technique was the uppercut. The uppercut is the second semicircular punching technique following the thrown hook. The detailed examination of the data sets of both groups of boxing subjects, the inexperienced and the experienced athletes, showed a normal distribution of the data using the Shapiro–Wilk test (*p* > 0.05).

The defensive position at the beginning of the test series, as well as before the individual test cycles, of the experienced group of test subjects was measured with an average rotation around the transverse axis of 46.71° (SD = 18.02°) and a supination of the fist of 86.36° (SD = 65.73°). In comparison to the experienced group, the defensive position of the non-experienced participants was taken with a rotation of 24.51° (SD = 11.29°) in the transverse axis and a supination of 98.09° in the sagittal axis. This corresponds to a mean difference of 22.2° between the two tested groups in the transverse axis (95% CI [−4.18°, 48.59°]) and −11.72° in the sagittal axis (95% CI [−69.79°, 46.35°]). The performed statistical analysis of the defensive position showed no statistically significant differences (*p* > 0.05).

**Figure 7:**
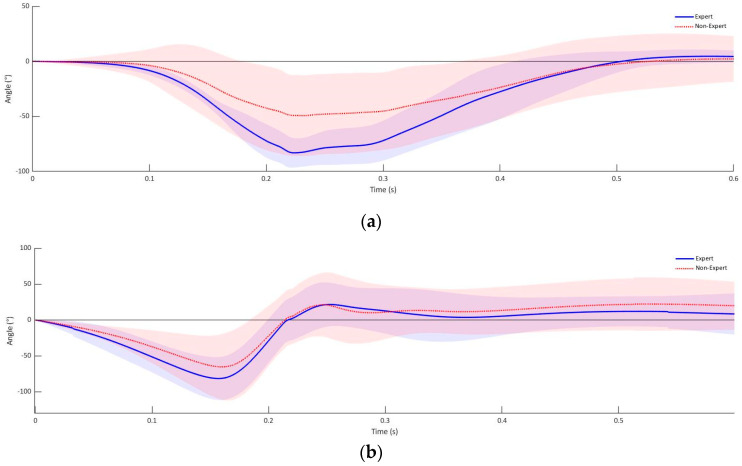

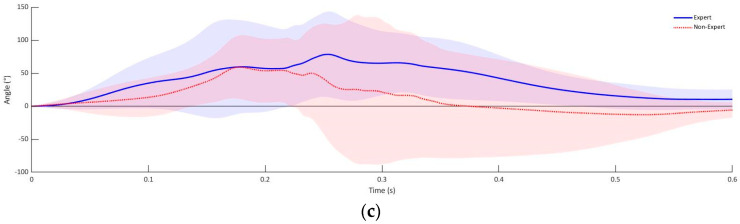
Fist rotation experienced athlete’s uppercut punch: (**a**) longitudinal, (**b**) transversal and (**c**) sagittal axis.

The rotation in three-dimensional space shown in Figure 7 shows that the rotation of the fist from the defensive position to the object to be hit is initiated by a simultaneous rotation around the longitudinal and transverse axis before a supination of the fist to the target is executed. At the point of time the fist makes contact with the object to be struck, the fist is displaced by −16.49° (SD = 7.43°) in the longitudinal axis from the defensive position. Likewise, the fist is tilted by 1.51° (SD = 9.15°) in the transverse axis and supinated by 59.53° from the defensive position in the experienced group of subjects (Figure 7). The rotation of the fist at the target in the non-expert group is rotated by −3.9° (SD = 6.95°) in the longitudinal axis, 1.53° (SD = 7.08°) in the transverse axis and supinated by 57.12° (SD = 12.75°) (Figure 7).

The investigation shows no statistically significant difference between the experienced and inexperienced test group in the rotation from the defensive position to the targeting object, around the longitudinal axis (*p* = 0.24) and the transverse axis (*p* = 0.9) as well as in the rotation around the sagittal plane of the fist between the two tested groups with (*p* = 0.94).

Analysis of the fist rotation for the retracted defensive position shows a mean deviation of 4.18° (SD = 10.28°) in the longitudinal axis, 5.18° (SD = 9.12°) in the transverse axis and 2.94° (SD = 5.05°) in the sagittal axis of the experienced group of subjects (Table 5). In comparison, the inexperienced group showed a larger mean difference. The assumed defensive position after the executed stroke showed a deviation from the first defense positioning prior to impact of −26.85° (SD = 27.4°) in the longitudinal axis, 34.89° (SD = 37.48°) in the transverse axis and −11.24° (SD = 20.53°) in the sagittal axis (Table 5). The results presented show a statistically significant difference between the two groups of subjects in terms of the deviation between the defensive position before and after the blow, in the longitudinal axis (*p* = 0.001), transverse axis (*p* = 0.009) as well as the sagittal axis (*p* = 0.02).

The total duration of the uppercut stroke was on average 385 ms (SD = 65 ms) in the experienced group of subjects. In comparison, the time of execution in the inexperienced group of subjects was measured with a mean difference of 68 ms and a total duration of 453 ms (SD = 60 ms). In a detailed analysis of the three defined impact phases, the experienced test subjects’ impact required an average of 71 ms (SD = 36 ms) from the defensive position to impact. The fist was in contact with the targeting object for a total of 143 ms (SD = 34 ms). The retraction phase back into the defense position was measured with 171 ms (SD = 33 ms). For the non-experienced group, the average time required for the throw phase was 83 ms (SD = 29 ms), for the contact period 163 ms (SD = 23 ms) and for the retraction phase 204 ms (SD = 41 ms) (Table 6). The investigation of significant effects (Table 7) shows a significant difference in both the absolute impact time (*p* = 0.01) and the duration of the retraction phase (*p* = 0.04) between the experienced and non-experienced group. No statistically significant differences were detected for the first and second stroke phase of the throw (*p* = 0.39) and the contact period (*p* = 0.10).

In addition to the investigation of the technical orientation variables of the fist in three-dimensional space, further punch variables between experienced and non-experienced subject groups were collected (Table 6). The results displayed in Table 6 show the mean punch forces and punch velocities achieved of the four tested punching techniques for the experienced and non-experienced group of test participants. Significant differences in the maximum achieved punch force for the hook, jab and uppercut technique were observed for the experienced group of subjects compared to the non-experienced group of participants. For the three punch types, the experienced group of test persons performed a mean of 1322.66 N (SD = 561.66 N) greater maximum punch force than the test persons with lesser boxing experience. No significant differences were observed when comparing the maximum punch velocities between experienced and non-experienced participants.

## 4. Discussion

As described in detail above, the athletes sporting performance depends to a large extent on the technical execution of the athletic motion to achieve maximum effectiveness of the physical performance in attacking as well as defensive situations as described by McGarry and colleagues in 2013 [1].

A variety of different measurement methods have been used to analyze biomechanical impact parameters in martial arts. One focus of previous measurement methods was the use of inertial sensors [63]. These sensors do not allow for a comprehensive examination of punching parameters to evaluate punching effectiveness and efficiency. To address this gap, a comprehensive boxing performance monitoring system was used, including force-sensing resistors and inertial sensors.

For the application of the piezoresistive sensors, a special focus of the development was on the sensor properties to avoid creep behavior as well as large hysteresis. During the development, the sensor system was designed with a hysteresis of only 1.91% and a reduction of the sensor creep by 99.99% after 0.28 s. Due to these properties, the developed sensor system showed excellent results during the validation and were perfectly suited for further research.

A problem with the use of inertial sensors constitutes the phenomenon of the gimbal lock. To circumvent this problem, the use of Euler angles was avoided, and the angles were determined by use of quaternions. For this purpose, a Madgwick quaternion filter was programmed on the microelectronics. The Madgwick sensor fusion filter is based on a quaternion representation. This has the advantage of avoiding the limitations observed with Euler angle representations, such as singularity effects, while determining the three-dimensional orientation of the fist in space when throwing a punch. The Madgwick sensor fusion filter was applied as it exhibits a reduced implementation complexity, that is particularly important for limited power and processing applications, as well as providing a good handling for low and high sampling rates as it is necessary in the developed sensor system [64,65]. The Madgwick sensor fusion filter combines the three sensor output signals of the tri-axis accelerometer, gyroscope and magnetometer to form a comprehensive 3D measurement system. In addition to the fusion of the three sensor signals, the Madgwick filter contains a compensation of error signals caused by magnetic distortion. For the gyroscope angle determination, the acceleration and magnetometer sensor outputs are used by an optimized and analytically derived gradient descent algorithm. This enables the direction of the gyroscope measurement error to be determined exactly by a quaternion derivative.

The purpose of the experimental research was to present a first field investigation by use of the developed sensor system and to highlight the possibilities of the measurement parameters generated by the sensor system to be compared with the existing scientific literature. Furthermore, the study builds on existing scientific insights into the technical execution of boxing and martial arts striking techniques. For this purpose, a technical comparison of athletes with different levels of experience regarding punch execution and fist rotation in three-dimensional space for the four main punching techniques of the jab, cross, hook and uppercut was conducted.

To the authors’ knowledge, this is the first experimental study that analyzes the technical aspects of the four main punching techniques, with a specific observation of the fist orientation in three-dimensional space from the defense orientation to the impact rotation and return of the fist, by use of a wearable boxing sensor system.

The statistical results of the ANOVA data analysis demonstrated significant performance differences between the experience level, the performed stroke technique, as well as the interaction between experience level and stroke technique.

The results of the technical analysis of fist orientation in three-dimensional space have shown that the fist orientation taken at the beginning of each punch in the defensive position differs between the two groups of test persons of experienced and non-experienced athletes. The results show that the defensive position of the group of subjects, classified as experts, is taken with an average rotation of 56.42° (SD = 6.82°) in the transverse axis and 104.94° (SD = 12.59°) in the sagittal axis. The uppercut stroke technique showed the greatest deviation (9.71°) from the mean defensive position with 46.71° (SD = 18.02°) compared to the cross, hook and jab. The average defensive position of the non-experienced athletes was shown with a deviation of −4.42° from the experienced group of subjects. The examination revealed no significant but tendential deviations between the two subject groups regarding their fist orientation in the defensive position of the cross, jab and uppercut punch. A statistically significant difference of 15.83° was observed on average in the transverse axis of the hook punch defensive position.

Greater statistically significant results were shown in the differences in the rotation from the defensive position to the targeting object. The results demonstrated a statistically significant difference in the rotation of the fist in the sagittal axis of 35.01° (SD = 7.34°) on average between experienced and non-experienced athletes in each of the four striking techniques performed. The pronation of the fist in the direction of the object to be hit is of particular importance for the optimal impact area of the fist, as described by Arus [66], that the palm is facing downwards to hit the target with the second to fourth heads of the metacarpals and the metacarpophalangeal (MCP) joints.

The analysis of fist orientation in three-dimensional space has furthermore demonstrated that the rotation of the fist is initiated prior to the acceleration of the fist towards the target object. The initial rotation starts on average 0.1 to 0.2 s before the actual throw phase is initiated.

In the third phase of action, following the executed impact, returning the fist to the defensive position, it is shown that the group of test persons of the experienced athletes demonstrated an average deviation from the initial defensive position of 2.03° (SD = 5.1°) in the longitudinal axis, −0.79° (SD = 4.69°) in the transverse axis and −0.09° (SD = 3.24°) in the sagittal axis. With a significant larger deviation, the defensive position of the non-experienced group of subjects was taken with −11.33° (SD = 13.51°) in the longitudinal axis, 7.46° (SD = 19.52°) in the transverse axis and −3.67° (SD = 7.46°) in the sagittal axis. A maximum average deviation of −26.85° (SD = 27.4°) up to 34.89° (SD = 37.48°) was observed in the uppercut punching technique. The retracted orientation of the defensive position revealed a significantly higher technical reproducibility for the experienced group of subjects compared to the non-experienced group.

Beyond this, the present study sought to evaluate the punch speed and punch force and compare experienced and non-experienced boxers. Furthermore, the time period of the three defined punching phases was examined. The analysis of sport-specific time-motion variations is a non-invasive method of performance diagnostics for the examination of performance characteristics and movement patterns [67].

The investigation of the mean and the maximum punching speeds achieved before impact has shown that no significant differences emerged between the groups of experienced and non-experienced participants or between the punching techniques within a subject group. A detailed examination of the results reveals that the group of experienced participants showed a greater tendency of punching speed in all measurements of maximum and mean punching results for the four punching techniques executed. These results are consistent with the findings of Whiting et al. [49] that more experienced athletes exhibit a greater overall punch speed than athletes with less experience.

The punching techniques of the jab and cross showed an equal maximum speed of 7.88 m/s in the group of experienced test persons. In addition, the mean fist velocity of 6.6 m/s in the cross technique showed consistency with the published measurement results of Whiting [49] as well as with the results published by Baitel and Deliu [68]. Furthermore, the cross has shown the shortest mean contact time of both groups of subjects for all punching techniques performed.

In the comparison of the two semicircular punching techniques of the rear hand hook and the uppercut, the rear hand hook revealed a 0.12 m/s moderately greater maximum punch speed of 6.93 m/s (SD = 0.93 m/s), than the uppercut with 6.81 m/s (SD = 0.89 m/s). These measurement results show a considerable deviation from results of previous studies [46,49]. According to the literature, the hook punching technique has achieved a higher stroke speed than the jab or cross. The greater punch speed is based on the fact that the hook stroke generates a greater range of movement due to shoulder flexion and adduction than it can be achieved with the jab or cross, that is mainly executed via the elbow extension.

The extended acceleration distance is, moreover, the main factor in the significantly longer mean throw time of the hook. The two tested groups demonstrated a threefold higher duration of the throw phase compared to the straight punching techniques of the cross and jab. Whiting et al. [49] and Piorkowski et al. [46] have also demonstrated a greater punch execution time before impact for the hook compared to jab and cross, albeit with less significance.

Despite a lower striking speed, the two semicircular striking techniques of the rear hand hook with 4177.47 N (SD = 1155.04 N) and the uppercut with 3851.03 N (SD = 768.92 N) show significantly higher striking forces compared to the straight punches of the cross and jab. This result leads to the assumption that the experienced athletes transferred a higher effective mass into the punch. The investigation of the effective mass used, provides a further point of investigation for follow-up studies to extend the range of investigation in martial arts between experienced and non-experienced athletes. The mean punch forces achieved with the jab (1383 N, SD = 234.81 N), the cross (1918.82 N, SD = 787.49 N) and rear hand hook (1949.08 N, SD = 395.27 N) for the experienced subject group displayed similar results to the study by Lenetsky and colleagues [39].

The longest total mean punch time from the initial fist movement to target and return to the defensive position was measured in the jab for both groups of non-experienced 485 ms (SD = 98 ms) and experienced subjects at 523 ms (SD = 63 ms). In contrast, the shortest duration of the mean throw time was measured in the uppercut technique with 71 ms (SD = 36 ms) in the experienced group and 83 ms (SD = 29 ms) in the non-experienced group of subjects. The short mean throw time can be explained mainly by the shorter distance to the object of impact. Both groups of subjects performed the uppercut technique with the shortest distance to the object compared to the cross, jab and rear hand hook technique.

Furthermore, the punch impact was determined to further evaluate the punch effectiveness. The results show no statistically significant differences between the experience levels. The results also indicate that, due to the longer contact time of the inexperienced subjects, a higher impact was measured for the two semicircular punching techniques. These punching techniques are considered more demanding punching techniques, making the use of the punch impulse an unreliable variable for determining punching effectiveness.

Results in the sport of boxing and martial arts were obtained by comparing experienced and non-experienced test subjects regarding their technical execution of the four main punching techniques tested. The experiment undertook data collection during a normal training session on a punching bag. At no point in time in this study were data collected in a competition-specific situation as it is presented by a sparring training or a regular boxing match. This type of competition situation does not allow the athletes to focus on a single maximum stroke, but rather is carried out purely on the basis of the context, resulting in a deviation in maximum stroke forces, speed and technical-temporal movement sequences. In addition, only single maximal strokes were performed in the current study. The comparison to punching combinations would provide further insights, as the study by Piorkowski et al. [46] has shown that a significant difference between punching combinations and single maximal punches could be measured in terms of contact speed.

Based on the results of Piorkowski et al. [46], follow-up studies to examine punch combinations, with regard to the temporal sequence of the individual punch phases as well as the retraction orientation of the fist, would be extremely useful.

For further investigation, a third group of subjects should be considered in a follow-up study. For this purpose, the level of experience should be extended and athletes with international experience should be added. Furthermore, another potential follow-up could examine the technical execution of the tested strokes in different situations, such as competition, in order to be able to compare the performance outcome with the two previous groups of experience and to highlight potential movement patterns executed. Finally, with regard to the selection of participants regarding their level of experience, it is suggested that a more homogeneous group of subjects could be selected for the individual experience groups to help identify a clear distinction between movement patterns of subjects according to ability and level of experience.

## 5. Conclusions

According to the results, the research shows statistically significant differences in the technical execution between experienced and non-experienced subjects in the four main punching techniques of the jab, cross, rear hand hook and uppercut (Table 7).

The significant results can be used as a starting point for obtaining objective data to create a technical model and reference criteria to enable athletes to optimize punch effectiveness and efficiency by the help of data-based punch models. The possibility of three-dimensional analysis of the stroke trajectory demonstrates the possibility of conducting in field investigations for motion analysis, detached from laboratory requirements. The analysis of the trajectory in three-dimensional space shows the possibility to replace a camera system to a certain extent in order to display the hand trajectory and punch acceleration in three dimensions. Boxing and martial arts are defined by specific movement patterns that are not analyzed in competition. The developed monitoring system makes it possible to investigate these punching movements in the field and to determine the punching effect from the obtained and analyzed information.

Furthermore, the presented results show a concordance with the results of previous publications in the areas of punch force, punch speed and punch duration assessment.

The developed system has been able to demonstrate its applicability in the conducted field study and thus enables further research in the field of boxing and martial arts to expand the current biomechanical information available. The knowledge gained from the experimental data can offer coaches and athletes a tool for analyzing the requirements of a specific punching movement pattern with the help of a novel boxing monitoring system. The results of this study can be used to apply technological data-based analysis for talent identification and promotion in martial arts, by a system as it is demonstrated in this work. Coaches and performance support centers in particular can thus benefit from such a measurement system, with which the technical performance of boxing strokes can be measured and potential technique correction can be made in the interests of the athlete by objective data.

## Figures and Tables

**Table 1:** Subject characteristics of the experienced and non-experienced groups of boxing athletes.

	Experienced (*n* = 11)	Non-Experienced (*n* = 20)
Age (years) ^1^	26.29 ± 4.54	21.67 ± 2.46
Height (cm) ^1^	178.86 ± 6.57	179.27 ± 9.76
Body mass (kg) ^1^	79.43 ± 9.31	75.92 ± 8.15
Experience (years) ^1^	7.43 ± 3.34	0.36 ± 0.44

^1^ Values are means ± SD.

**Table 2:** Difference in angular orientation between the initial and retracted defensive position cross punch.

	Experienced	Non-Experienced
Longitudinal rotation ^1^	−4.24° ± 3.85°	−4.95° ± 17.36°
Transversal rotation ^1^	−1.92° ± 4.33°	−2.51° ± 7.79°
Sagittal rotation ^1^	−0.17° ± 6.42°	6.1° ± 14.93°

^1^ Values are means ± SD.

**Table 3:** Difference in angular orientation between the initial and retracted defensive position hook punch.

	Experienced	Non-Experienced
Longitudinal rotation ^1^	−9.78° ± 7.16°	−25.19° ± 30.94°
Transversal rotation ^1^	−0.26° ± 2.45°	7.10° ± 19.62°
Sagittal rotation ^1^	−4.56° ± 12.38°	−2.26° ± 23.1°

^1^ Values are means ± SD.

**Table 4:** Difference in angular orientation between the initial and retracted defensive position jab punch.

	Experienced	Non-Experienced
Longitudinal rotation ^1^	6.14° ± 8.47°	−2.2° ± 15.43°
Transversal rotation ^1^	−6.16° ± 4.31°	−9.63° ± 11.47°
Sagittal rotation ^1^	1.4° ± 2.8°	−7.24° ± 18.34°

^1^ Values are means ± SD.

**Table 5:** Difference in angular orientation between the initial and retracted defensive position uppercut punch.

	Experienced	Non-Experienced
Longitudinal rotation ^1^	4.18° ± 10.28°	−26.85° ± 27.4°
Transversal rotation ^1^	5.18° ± 9.12°	−34.89° ± 37.48°
Sagittal rotation ^1^	2.94° ± 5.05°	−11.24° ± 20.53°

^1^ Values are means ± SD.

**Table 6:** Punch variables of the four tested punching techniques.

	Cross	Rear Hand Hook	Jab	Uppercut
Experienced				
Total mean punch time (ms)	402 ± 65	441 ± 104	523 ± 63	385 ± 65
Mean throw time (ms)	111 ± 41	91 ± 50	117 ± 25	71 ± 36
Mean contact time (ms)	122 ± 18	141 ± 29	144 ± 27	143 ± 34
Mean retraction time (ms)	169 ± 41	168 ± 97	262 ± 42	171 ± 33
Peak fist velocity (m/s)	7.88 ± 1.0	6.93 ± 0.9	7.9 ± 0.9	6.8 ± 0.9
Mean fist velocity (m/s)	6.6 ± 0.9	5.87 ± 0.9	6.3 ± 0.9	5.40 ± 0.8
Peak force (N)	3149.1 ± 741.3	4177.5 ± 1155	3167.8 ± 676.2	3851.0 ± 768.9
Mean force (N)	1918.8 ± 787.5	1946.2 ± 720.6	1383 ± 234.8	1949.1 ± 395.3
Punch impulse (N·s)	223.2 ± 62.4	277.3 ± 79.2	189.4 ± 22.5	236.5 ± 83.8
Non-experienced				
Total mean punch time (ms)	450 ± 104	479 ± 117	485 ± 98	453 ± 60
Mean throw time (ms)	102 ± 37	72 ± 233	135 ± 42	83 ± 29
Mean contact time (ms)	118 ± 25	212 ± 198	138 ± 28	163 ± 23
Mean retraction time (ms)	235 ± 79	186 ± 83	212 ± 75	204 ± 41
Peak fist velocity (m/s)	7.6 ± 1.4	6.6 ± 1.2	6.9 ± 1.1	6.34 ± 0.8
Mean fist velocity (m/s)	5.7 ± 0.9	5.8 ± 1.0	6.0 ± 1.1	5.03 ± 0.9
Peak force (N)	2936.4 ± 662.1	2206.9 ± 646.7	2154.6 ± 503.9	2867.16 ± 540.1
Mean force (N)	1756.9 ± 752.8	1722.0 ± 405.2	1372.4 ± 415.2	1791.9 ± 607.6
Punch impulse (N·s)	215.3 ± 64.7	387.7 ± 46.4	186.5 ± 47.9	295.8 ± 86.3

Values are means ± SD.

**Table 7:** Presentation of the significant punch type results.

Punch Technique (Expert vs. Novice)	Significant Variable	*p*
Cross	Rotation around the sagittal axis from the defensive position to target	<0.001
Hook	Defensive position of the transverse axis	=0.004
Jab	Rotation around the transverse axis to target	=0.02
	Rotation around the sagittal axis to target	<0.001
	Absolute impact time	=0.01
	Duration of the retraction phase	=0.04
Uppercut	Deviation between the defensive position before and after the blow longitudinal axis	=0.001
	Deviation between the defensive position before and after the blow transverse axis	=0.009
	Deviation between the defensive position before and after the blow sagittal axis	=0.02
	Absolute impact time	=0.01
	Retraction time	=0.04

Note. A 95% Confidence Interval was applied.

## Data Availability

The data presented in this study are available on request from the corresponding author. The data are not publicly available due to further research work. Data will be made available in the near future.
